# Positive association between *ALDH2* rs671 polymorphism and essential hypertension: A case-control study and meta-analysis

**DOI:** 10.1371/journal.pone.0177023

**Published:** 2017-05-04

**Authors:** Yinyin Wu, Juntao Ni, Xiao Cai, Fuzhi Lian, Haiyan Ma, Liangwen Xu, Lei Yang

**Affiliations:** School of Medicine, Hangzhou Normal University, Hangzhou, Zhejiang, China; University of Adelaide, AUSTRALIA

## Abstract

**Background and objective:**

Several studies have been conducted to examine the association between aldehyde dehydrogenase 2 family (*ALDH2*) rs671 polymorphism and essential hypertension (EH). However, the results remain inconsistent. This study aimed to clarify the association between *ALDH2* rs671 polymorphism and EH susceptibility.

**Methods:**

One thousand and ninety-four cases and 1236 controls who were ethnic Han Chinese were collected for this population-based case-control study. A meta-analysis was performed to calculate the pooled odds ratio and 95% confidence interval, using allele contrast, dominant, recessive, and co-dominant models using fixed or random-effect models.

**Results:**

Significant differences were observed between EH cases and controls at the level of both genotype (χ^2^ = 6.656, P<0.05) and alleles (χ^2^ = 6.314, P<0.05). An additional meta-analysis using 4204 cases and 5435 controls established that rs671 was significantly associated with EH (P<0.00001).

**Conclusion:**

The results of our case-control study and meta-analysis showed that there is a significant association between *ALDH2* rs671 polymorphism and EH susceptibility. In addition, the results of the breakdown analysis by gender suggest a male-specific association between the *ALDH2* rs671 polymorphism and EH.

## Introduction

Essential hypertension (EH) is a critical cardiovascular risk factor that may lead to stroke, coronary heart disease, diabetes and other diseases [1]. In China, EH is the top mortality risk factor among the population aged >40 years [2]. According to the Inter-ASIA Program, the prevalence of EH among Chinese adults was 27.2% in 2001, which means there were~130 million people with EH nationwide. Age-specific prevalence of EH was 10.7%, 26.8%, 38.9% and 50.2% for women and 17.4%, 28.2%, 40.7% and 47.3% for men among those aged 35–44, 45–54, 55–64 and 65–74 years, respectively [3]. The incidence of EH increased annually, impairing people’s health seriously.

EH is a complex disease, on which both environmental factors and genetic factors have an important impact [4]. Alcohol consumption is often considered as an important environmental factor, amenable to lifestyle modification, in the development of hypertension and cardiovascular disease [5]. Genetic factors account for 25%–65% of the blood pressure variation among individuals [6]. And to date very few genetic factors are understood [7]. It has also been suggested that genetic variation in alcohol-metabolizing enzymes affects the development of hypertension via their regulation of drinking behavior or sensitivity to alcohol [8].

Aldehyde dehydrogenase-2 (*ALDH2*) is located on chromosome 12q24 and is one of the key enzymes involved in ethanol metabolism [9].rs671 is a single nucleotide polymorphism (SNP) in exon 12 within *ALDH2*. The point mutation of base G to A changes the position of amino acid residue 504 from glutamic acid to lysine, resulting in the decrease of enzyme activity [10, 11]. The inactive *ALDH2* generally inhibits individuals from heavy drinking, leading to acetaldehydemia and alcohol flushing responses[12]. Therefore, this SNP of *ALDH2* may be associated with EH.

Several studies have been conducted to investigate the relationship between rs671 polymorphism and EH [13–16]. However, there were no consistent results. Though there were several meta-analyses that studied the relationship between rs671 polymorphism and EH, most of those previous studies only reported the odds ratio (OR) with the 95% confidence interval (CI) for the GG genotype compared with the AG+AA genotype [8,17–19]. Thus, we conducted a population-based case-control study, and then performed a comprehensive meta-analysis to further explore the association between rs671 polymorphism and EH under all genetic models.

## Materials and methods

### Subjects

This population-based case-control study included 1094 EH cases and 1236 healthy controls that participated in routine health examination at local community health centers between April and July 2013 in Yinzhou District, Ningbo City, Zhejiang Province, China. The cases were recruited if they met the following criteria: (1) systolic blood pressure (SBP) ≥140 mm Hg and/or diastolic blood pressure (DBP) ≥90 mm Hg when taking no antihypertensive medication; (2) previously diagnosed with EH; (3) taking antihypertensive medication; and (4) aged 40–70 years. All subjects were free from secondary hypertension, diabetes mellitus, renal disease, thyroid disease or a history of cancer, and were ethnic Han Chinese. The study protocol was approved by the Medical Ethical Committee of the Affiliated Hospital of Hangzhou Normal University. After written informed consent was obtained from the subjects, a face-to-face interview was conducted to collect information, including demographic (e.g. sex and age) and lifestyle (e.g. smoking and drinking) data, and 5-ml blood samples were collected. Those who smoked at least one cigarette per week were defined as current smokers. Those who drank at least once per week were defined as current drinkers. Thus those former smokers and never smokers were classified as non-smokers, and those former drinkers and never drinkers were defined classified as non- drinkers. Besides, weight and height were also measured using standardized methods during the interview and body mass index (BMI) was calculated by the standard formula [weight (kg)/height^2^ (m^2^)].

### DNA extraction, and SNP genotyping

Genomic DNA was isolated from peripheral blood samples using TIANamp Blood DNA Kits (Tiangen Biotech, Beijing, China) and was stored at −80°C. Genotyping of *ALDH2* rs671 polymorphism was carried out using the polymerase chain reaction–ligase detection reaction (PCR-LDR) method (Generay Biotech Company, Shanghai, China). The primer sequences were 5'-TCAAATTACAGGGTCAACTGC-3' (forward) and 5'-AGCCACCAGCAGACCCTCAA-3' (reverse). The probe sequences were TTTTGAGTACGGGCTGCAGGCATACACTA (TA), TTTTTTTGAGTACGGGCTGCAGGCATACACTG (TG), and -P-AAGTGAAAACTGTGAGTGTGGGACCTTT-FAM- (TR). The PCRs were performed in an ABI Prism 7000 Sequence Detection System (Foster City, CA, USA) in a total volume of 15μl, including 1 μl genomic DNA, 1.5 μl 10× PCR buffer, 1.5 μl MgCl_2_, 0.3 μl dNTPs, 0.15 μl each primer, and 0.2 μl Taq DNA polymerase. The PCR was performed as follows: an initial melting step of 3 min at 94°C, 35 cycles of denaturation for 15 s at 94°C, annealing for 15 s at 55°C and extension for 30 s at 72°C, followed by 3 min final extension at 72°C.The ligation reaction for each PCR product was carried out with a total volume of 10 μl, including 3 μl PCR product, 1 μl 10×Taq DNA ligase buffer, 5 U Taq DNA ligase, and 0.01 μl each discriminating probe. The LDR was performed as follows:30 cycles at 94°C for 30 s and 56°C for 3 min. After the LDR, 1 μl LDR product was mixed with 8 μl loading buffer, and was melted for 3 min at 95°C.The mixture was then analyzed on the ABI3730xl platform. Ten percent of the samples were randomly selected and genotyped repeatedly for quality control, and the concordance was 100%.

### Statistical analysis for case-control study

Differences in the distribution of demographic characteristics and genotypes of *ALDH2* rs671 polymorphism between the cases and controls were tested using the χ^2^ test. Whether the genotype distribution was in Hardy–Weinberg equilibrium (HWE) among the controls was tested using goodness-of-fit χ^2^ test. The associations between *ALDH2* rs671 polymorphism and EH risk were evaluated using unconditional logistic regression for crude odds ratio (OR) with the 95% confidence interval (CI) and adjusted OR with 95% CI. The statistical analyses were performed using SAS version 9.1 (SAS Institute, Cary, NC, USA). P<0.05 was considered statistically significant.

### Meta-analysis

The meta-analysis was reported on the basis of the Preferred Reporting Items for Systematic Review and Meta-analyses (PRISMA) guidelines [20].

### Literature search

We searched PubMed, Web of Science, and Embase up to March 2016 without language restrictions. The keywords were “aldehyde dehydrogenase-2”,”*ALDH2*” combined with “hypertension”. The search results were supplemented by screening references of the original articles and systematic reviews. E-mail was also used to contact study authors to obtain full text articles or missing data.

### Inclusion and exclusion criteria

Studies were included if they met the following criteria: (1) case-control studies; (2) studies assessed the association between *ALDH2* rs671 polymorphism and EH risk; (3) EH was diagnosed following the guidelines including SBP ≥140 mm Hg and/or DBP ≥90 mm Hg when taking no antihypertensive medication, previously diagnosed with EH, and taking antihypertensive medication; (4) the study had available allele or genotype frequencies for cases and controls. The exclusion criteria were: (1) articles were abstracts or reviews, or reported duplicate data; (2) no usable data; and (3) there was departure from HWE in genotype distribution of the control group or all subjects.

### Data extraction and quality assessment

Data were extracted from the included studies by two investigators independently. Disagreement was resolved by discussion or consultation with a third investigator. The following data were extracted from all obtained studies: first author’s name, publication year, country, ethnicity, study design, genotyping method, number of cases and controls, genotype and allele distributions of cases and controls, and HWE of cases and controls.

The quality of the included studies was evaluated through a checklist originated from Strengthening the Reporting of Genetic Association (STREGA) recommendations for reports on genetic association studies [21].

### Statistical analysis for meta-analysis

Goodness-of-fit χ^2^ test was used to test whether the genotype distribution was in HWE among the control group as well as all the subjects depending on the data available. The association between *ALDH2* rs671 polymorphism and EH risk was assessed by pooled ORs with 95% CIs under five genetic models (co-dominant model AA vs. GG, and AG vs. GG; dominant model AA/AG vs. GG; recessive model AA vs. AG/GG; and allele contrast A vs. G).Heterogeneity among studies was assessed by χ^2^ test-based Q-statistic and *I*^2^ statistic. If P<0.1 or *I*^2^>50%, the random-effects model was conducted; otherwise the fixed-effects model was adopted. Subgroup analysis was conducted with respect to country. Sensitivity analysis was performed to detect the individual effect of each study on the pooled ORs. Publication bias was tested by funnel plot. All statistical analyses were performed by Review Manager software (version 5.3, Cochrane Collaboration, Oxford, UK) and STATA (version 12.0, Stata Corporation, College Station, TX, USA). P<0.05 was considered statistically significant.

## Results

### Single-locus analysis

As shown in Table 1, the genotype frequencies of *ALDH2* rs671 polymorphism were in HWE among all the controls as well as when stratified by sex. The genotype frequencies of *ALDH2* rs671 polymorphism were 53.7% (GG), 40.3% (AG) and 6.0% (AA) in the cases, and 49.1% (GG), 43.0% (AG) and 7.9% (AA) in the controls, and there was a significant difference between cases and controls (P = 0.036). Logistic regression analyses showed that the *ALDH2* rs671 polymorphism was significantly associated with EH risk. When compared with individuals carrying GG genotype, those carrying AA or AA/GG genotype were at a lower risk of EH [AA vs. GG: OR (95% CI) = 0.67(0.46–0.96), AA/AG vs. GG: OR (95% CI) = 0.82(0.69–0.98)]. A further sex-stratified association showed that the rs671 polymorphism was significantly associated with EH risk in men [AA/AG vs. GG: OR (95% CI) = 0.76(0.58–0.98)] but not in women.

**Table 1:** Distribution of the *ALDH2* rs671 polymorphism, and drinking habit in the participants in the case-control study.

		Cases, n(%)	Controls, n(%)	Crude OR (95%CI)	P	Adjusted OR (95%CI) ^a^	P	P_HWE_
rs671								
Overall	GG	586(53.7)	606 (49.1)	1.00		1.00		0.218
	AG	440(40.3)	531(43.0)	0.86(0.72–1.02)	0.075	0.85(0.71–1.02)	0.084	
	AA	65(6.0)	98(7.9)	0.69(0.49–0.96)	0.027	0.67(0.46–0.96)	0.028	
	AA/AG	505(46.3)	629 (50.9)	0.83(0.71–0.98)	0.025	0.82(0.69–0.98)	0.029	
	AG/GG	1026(94.0)	1137(92.1)	1.00		1.00		
	AA	65(6.0)	98(7.9)	0.74(0.53–1.02)	0.063	0.72(0.5–1.02)	0.064	
	G	1612(73.9)	1743(70.6)	1.00	<0.001			
	A	570(26.1)	727(29.4)	0.85(0.77–0.93)				
Male	GG	267(52.8)	264(46.8)	1.00		1.00		0.398
	AG	206(40.7)	250(44.3)	0.81(0.63–1.05)	0.109	0.79(0.6–1.03)	0.081	
	AA	33(6.5)	50(8.9)	0.65(0.41–1.05)	0.076	0.61(0.37–1.03)	0.065	
	AA/AG	239(47.2)	300(53.2)	0.79(0.62–1)	0.052	0.76(0.58–0.98)	0.036	
	AG/GG	473(93.5)	514(91.1)	1.00		1.00		
	AA	33(6.5)	50(8.9)	0.72(0.45–1.13)	0.154	0.69(0.41–1.13)	0.142	
	G	740(73.1)	778(69.0)	1.00				
	A	272(26.9)	350(31.0)	0.82(0.68–0.99)	0.035			
Female	GG	319(54.5)	342(51.0)	1.00		1.00		0.344
	AG	234(40.0)	281(41.9)	0.89(0.71–1.12)	0.336	0.92(0.72–1.18)	0.497	
	AA	32(5.5)	48(7.2)	0.71(0.45–1.15)	0.164	0.72(0.43–1.21)	0.214	
	AA/AG	266(45.5)	329(49.0)	0.87(0.69–1.08)	0.207	0.89(0.7–1.13)	0.337	
	AG/GG	553(94.5)	623(92.8)	1.00		1.00		
	AA	32(5.5)	48(7.2)	0.75(0.47–1.19)	0.224	0.75(0.46–1.24)	0.262	
	G	872 (74.5)	965(71.9)	1.00				
	A	298(25.5)	377(28.1)	0.88(0.73–1.05)	0.139			
Drinking								
Overall	No	840(68.0)	755(69.1)	1.00		1.00		
	Yes	395(32.0)	338(30.9)	0.952(0.80–1.14)	0.583	1.109(0.89–1.38)	0.358	
Male	No	247(43.7)	231(45.5)	1.00		1.00		
	Yes	318(56.3)	277(54.5)	0.931(0.73–1.19)	0.563	1.250 (0.95–1.64)	0.108	
Female	No	593(88.5)	524(89.6)	1.00		1.00		
	Yes	77(11.5)	61(10.4)	0.897(0.63–1.28)	0.547	0.868(0.59–1.28)	0.472	

^a^ Adjusted for age, sex, BMI and smoking

Besides, the analysis of the distribution of drinking habit showed that there was no significant difference between cases and controls (P = 0.583), even after being adjusted by confounding factors (P = 0.358).

### Eligible articles for meta-analysis

**Fig 1:**
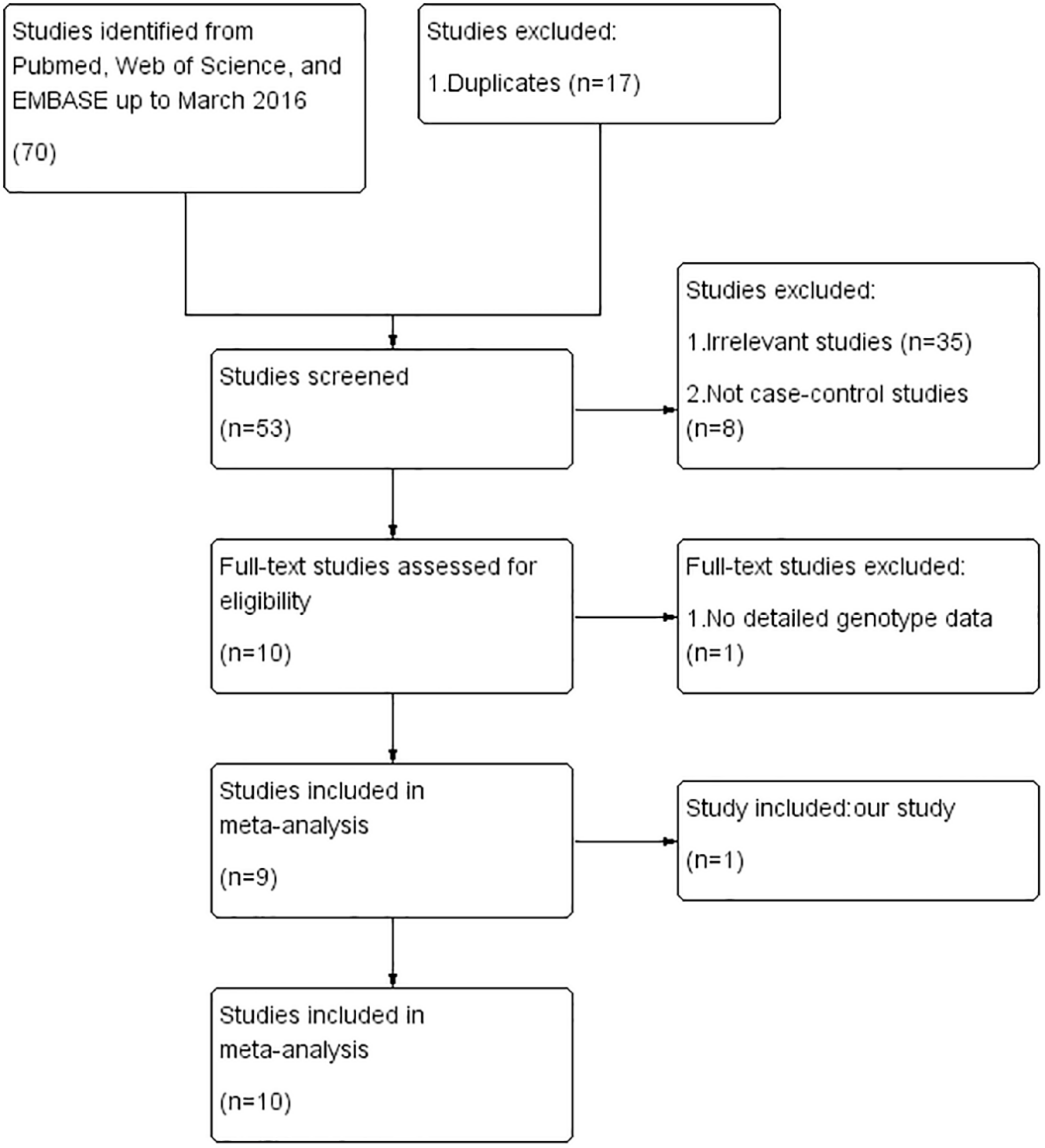
Flow diagram of article selection process for the *ALDH2* rs671 polymorphism and EH risk meta-analysis.

We found 70 potentially relevant publications by searching the existing literature databases. After applying the inclusion and exclusion criteria, 10 studies [13, 14, 22–28] with full text and available genotype data and our study were eligible for this meta-analysis. The detailed process of study selection is presented in Fig 1. The excluded articles and reasons were listed in S1 Text.

### Study characteristics

The detailed information of each study included in the meta-analysis is presented in Table 2. These studies were published between 2001 and 2016. Six studies were from Japan, and the remainder was from China. Five studies applied genotype data in men and women, three studies applied genotype data in all cases and controls, and two study were performed only in men. Eight studies applied AA, AG, and GG genotype data, and two only reported AA/AG and GG genotype data. Although no departure from HWE was observed among male or female controls in the Amamoto study, the genotype distribution among overall controls departed from the HWE, as well as Ma’s study. Thus, the genotype data among overall cases and controls from these two studies were removed. Besides, departure from HWE was also observed among male controls in the Yokoyama study as well as the Takagi study. Thus, the genotype data among male cases and controls from these two studies were also removed. The quality assessment of these included studies was provided in S1 Table.

**Table 2:** Characteristics of the included studies in the meta-analysis.

Author	Year	Country	Ethnicity	Genotyping method	Stratified	Case	Control	Case, n(%)				Control, n(%)				PHWE (control)
								GG	AG	AA	AA/AG	GG	AG	AA	AA/AG	
Ota	2016	Japan	Asians	PCR-RFLP	Male	199	1026	137 (68.8)	-	-	62 (31.2)	630 (61.4)	-	-	396 (38.6)	0.529
Ma	2015	China	Asians	DNA microarray	Overall	1210	1089	483 (39.9)	622 (51.4)	105 (8.7)	727 (60.1)	674 (61.9)	379 (34.8)	36 (3.3)	415 (38.1)	0.048
Nakagawa	2013	Japan	Asians	PCR-RFLP	Overall	123	321	74 (60.2)	-	-	49 (39.8)	171 (53.3)	-	-	150 (46.7)	>0.05
Yokoyama	2013	Japan	Asians	PCR-RFLP	Male	495	1407	433 (87.5)	62 (12.5)	0 (0.0)	62 (12.5)	1172 (83.3)	235 (16.7)	0 (0.0)	235 (16.7)	0.001
Wang	2013	China	Asians	PCR-LDR	Overall	1098	1021	668 (60.8)	373 (34.0)	57 (5.2)	430 (39.2)	560 (54.8)	396 (38.8)	65 (6.4)	461 (45.2)	0.653
Hasi	2011	China	Asians	TaqMan PCR	Overall	91	70	83 (91.2)	8 (8.8)	0 (0.0)	8 (8.8)	55 (78.6)	15 (21.4)	0 (0.0)	15 (21.4)	0.315
					Male	44	37	38 (86.4)	6 (13.6)	0 (0.0)	6 (13.6)	32 (86.5)	5 (13.5)	0 (0.0)	5 (13.5)	0.659
					Female	47	33	45 (95.7)	2 (4.3)	0 (0.0)	2 (4.3)	23 (69.7)	10 (30.3)	0 (0.0)	10 (30.3)	0.305
Hui	2007	Japan	Asians	TaqMan PCR	Overall	261	271	166 (63.6)	81 (31.0)	14 (5.4)	95 (36.4)	136 (50.2)	114 (42.1)	21 (7.7)	135 (49.8)	0.667
					Male	170	182	118 (69.4)	45 (26.5)	7 (4.1)	52 (30.6)	90 (49.5)	78 (42.9)	14 (7.7)	92 (50.5)	0.607
					Female	91	89	36 (39.6)	48 (52.7)	7 (7.7)	55 (60.4)	46 (51.7)	36 (40.4)	7 (7.9)	43 (48.3)	0.991
Amamoto	2002	Japan	Asians	PCR-RFLP	Overall	788	1247	395 (50.1)	342 (43.4)	51 (6.5)	393 (49.9)	584 (46.8)	564 (45.2)	99 (7.9)	663 (53.2)	0.020
					Male	312	437	161 (51.6)	134 (42.9)	17 (5.4)	151 (48.4)	174 (39.8)	217 (49.7)	46 (10.5)	263 (60.2)	0.071
					Female	476	810	234 (49.2)	208 (43.7)	34 (7.1)	242 (50.8)	410 (50.6)	347 (42.8)	53 (6.5)	400 (49.4)	0.071
Takagi	2001	Japan	Asians	TaqMan PCR	Overall	1540	2517	809 (52.5)	598 (38.8)	133 (8.6)	731 (47.5)	1227 (48.7)	1065 (42.3)	225 (8.9)	1290 (51.3)	0.778
					Male	773	1146	421 (54.5)	289 (37.4)	63 (8.2)	352 (45.5)	503 (43.9)	536 (46.8)	107 (9.3)	643 (56.1)	0.035
					Female	767	1371	388 (50.6)	309 (40.3)	70 (9.1)	379 (49.4)	724 (52.8)	529 (38.6)	118 (8.6)	647 (47.2)	0.130
Our study	2015	China	Asians	PCR-LDR	Overall	1091	1235	586 (53.7)	440 (40.3)	65 (6.0)	505 (46.3)	606 (49.1)	531 (43.0)	98 (7.9)	629 (50.9)	0.218
					Male	506	564	267 (52.8)	206 (40.7)	33 (6.5)	239 (47.2)	264 (46.8)	250 (44.3)	50 (8.9)	300 (53.2)	0.398
					Female	585	671	319 (54.5)	234 (40.0)	32 (5.5)	266 (45.5)	342 (51.0)	281 (41.9)	48 (7.2)	329 (49.0)	0.344

### Meta-analysis results

**Fig 2:**
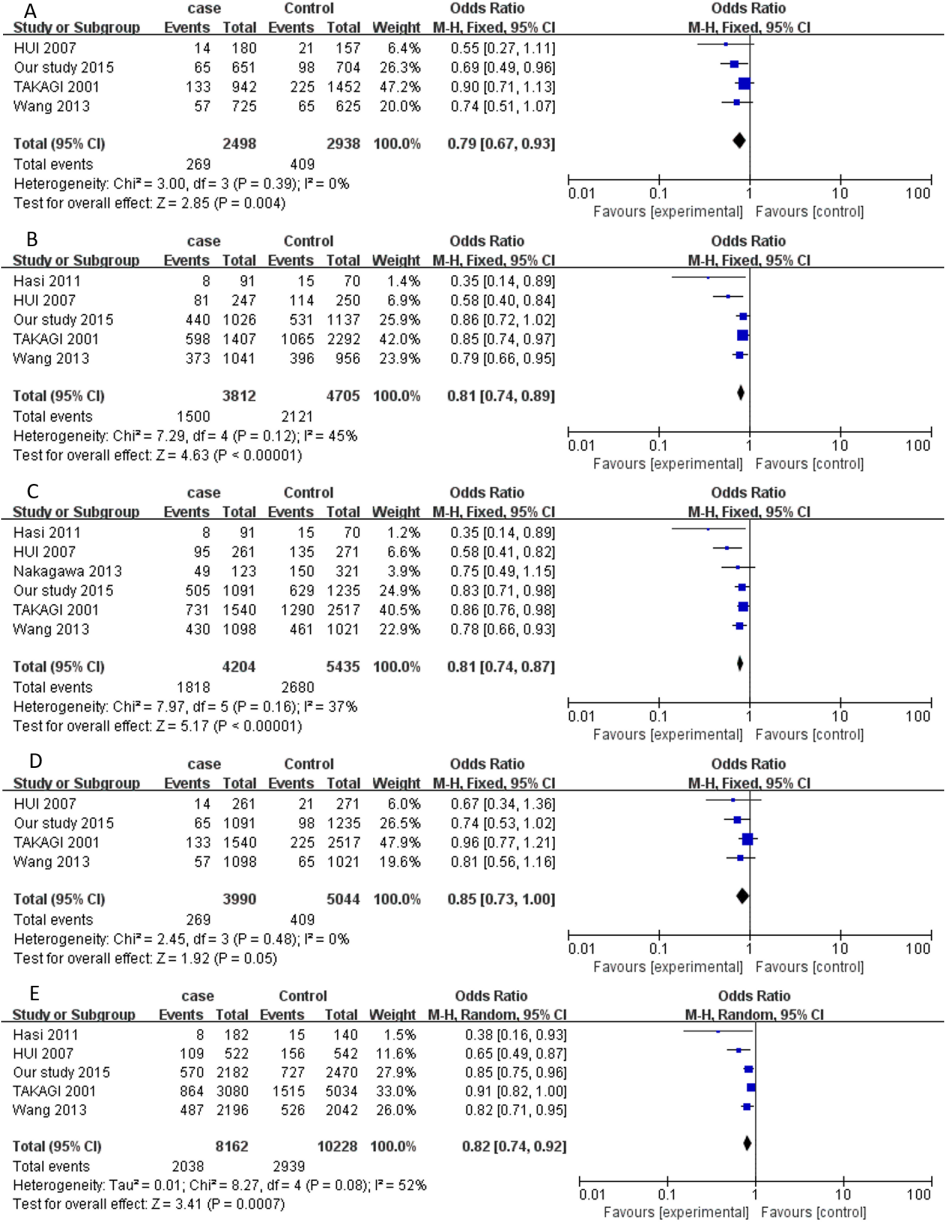
Forest plot of risk of EH associated with *ALDH2* rs671 polymorphism. (A) co-dominant model (AA vs. GG); (B) co-dominant model (AG vs. GG); (C) dominant model (AA/AG vs. GG); (D) recessive model (AA vs. AG/GG); (E) allelic contrast model (A vs. G).Error bars indicate 95% CI. Solid squares represent each study in the meta-analysis. Solid diamonds represent pooled OR.

After combining all qualified data, the total number of cases and controls were 8963 and 13 047, respectively, from eight eligible case-control studies. Overall, a significantly decreased risk was observed under four genetic models: co-dominant model AA versus GG (OR = 0.79, 95%CI = 0.67–0.93);co-dominant model AG versus GG (OR = 0.81, 95%CI = 0.74–0.89); dominant model AA/AG versus GG (OR = 0.81, 95%CI = 0.74–0.87); allelic contrast model A versus G (OR = 0.82, 95%CI = 0.74–0.92)] (Table 3, Fig 2). Subgroup meta-analysis by country indicated a significant association between rs671 polymorphism and EH risk in all genetic models for Chinese cases and controls. For Japanese cases and controls, there was a decreased risk of EH risk in the dominant model: AA/AG versus GG (OR = 0.75, 95% CI = 0.58–0.96).

**Table 3:** Meta-analysis of association between *ALDH2* rs671 polymorphism and EH risk in all participants.

Category	Subgroup	Genetic comparison	N^a^	OR (95% CI)	P ^b^	Test of heterogeneity	
						P, I^2^ (%)	Effect model
Overall		AA vs. GG	4	0.79 (0.67–0.93)	0.004	0.39, 0.00	F
		AG vs. GG	5	0.81 (0.74–0.89)	< 0.00001	0.12,0.45	F
		AA/AG vs. GG	6	0.81 (0.74–0.87)	< 0.00001	0.16,0.37	F
		AA vs. AG/GG	4	0.85 (0.73–1.00)	0.05	0.48, 0.00	F
		A vs. G	5	0.82 (0.74–0.92)	0.0007	0.08,0.52	R
Country	China	AA vs. GG	2	0.71 (0.55–0.91)	0.006	0.79, 0.00	F
		AG vs. GG	3	0.81 (0.72–0.92)	0.0009	0.17, 0.44	F
		AA/AG vs. GG	3	0.80 (0.71–0.90)	0.0001	0.20, 0.39	F
		AA vs. AG/GG	2	0.76 (0.60–0.98)	0.03	0.71, 0.00	F
		A vs. G	3	0.83 (0.75–0.91)	<0.0001	0.22, 0.34	F
	Japan	AA vs. GG	2	0.85 (0.69–1.07)	0.16	0.20, 0.40	F
		AG vs. GG	2	0.73 (0.51–1.05)	0.09	0.05, 0.73	R
		AA/AG vs. GG	3	0.75 (0.58, 0.96)	0.02	0.10, 0.57	R
		AA vs. AG/GG	2	0.93 (0.75–1.15)	0.51	0.34, 0.00	F
		A vs. G	2	0.79 (0.58–1.08)	0.14	0.03, 0.78	R

^a^ Number of studies

^b^ P for OR

Co-dominant model; dominant model; recessive model; allelic contrast model

After stratification by sex, the association between rs671 polymorphism and EH risk remained significant in all genetic models overall and in Japanese male subjects (Tables 4 and 5). We only found a significant association with the allelic contrast model among Chinese male subjects (A vs. G, OR = 0.82, 95% CI = 0.68–0.99). No significant association between rs671 polymorphism and EH risk was found in women.

**Table 4:** Meta-analysis of association between *ALDH2* rs671 polymorphism and EH risk in male participants.

Category	Subgroup	Genetic comparison	N^a^	OR (95%CI)	P value ^b^	Test of heterogeneity	
						P, I^2^(%)	Effect model
Overall		AA vs. GG	3	0.51 (0.36–0.72)	0.0001	0.36, 0.30	F
		AG vs. GG	4	0.70 (0.58–0.83)	< 0.0001	0.12, 0.48	F
		AA/AG vs. GG	5	0.64 (0.48–0.85)	0.002	0.10, 0.52	R
		AA vs. AG/GG	3	0.60 (0.43–0.84)	0.003	0.56, 0.00	F
		A vs. G	4	0.72 (0.63–0.82)	< 0.00001	0.12, 0.49	F
Country	China	AA vs. GG	1	0.65 (0.41–1.05)	0.08	-	F
		AG vs. GG	2	0.82 (0.64–1.05)	0.12	0.75, 0.00	R
		AA/AG vs. GG	2	0.79 (0.63–1.01)	0.06	0.71, 0.00	F
		AA vs. AG/GG	1	0.72 (0.45–1.13)	0.15	-	F
		A vs. G	2	0.82 (0.68–0.99)	0.04	0.74, 0.00	F
	Japan	AA vs. GG	2	0.39 (0.24–0.65)	0.0003	0.94, 0.00	F
		AG vs. GG	2	0.56 (0.38–0.84)	0.005	0.14, 0.55	R
		AA/AG vs. GG	3	0.61 (0.50–0.74)	< 0.00001	0.18, 0.42	F
		AA vs. AG/GG	2	0.50 (0.30–0.81)	0.005	0.93, 0.00	F
		A vs. G	2	0.62 (0.52–0.75)	< 0.00001	0.20, 0.38	F

^a^ Number of studies

^b^ P for OR

Co-dominant model; dominant model; recessive model; allelic contrast model

**Table 5:** Meta-analysis of association between *ALDH2* rs671 polymorphism and EH risk in female participants.

Category	Subgroup	Genetic comparison	N^a^	OR (95%CI)	P ^b^	Test of heterogeneity	
						P, I^2^(%)	Effect model
Overall		AA vs. GG	4	1.01 (0.81–1.26)	0.94	0.43, 0.00	F
		AG vs. GG	5	1.02 (0.79–1.31)	0.90	0.01, 0.68	R
		AA/AG vs. GG	5	1.01 (0.78–1.30)	0.94	0.01, 0.70	R
		AA vs. AG/GG	4	0.99 (0.79–1.23)	0.91	0.61, 0.00	F
		A vs. G	5	1.00 (0.83–1.21)	0.99	0.02, 0.66	R
Country	China	AA vs. GG	1	0.71 (0.45–1.15)	0.16	-	F
		AG vs. GG	2	0.35 (0.04–2.89)	0.33	0.008, 0.86	R
		AA/AG vs. GG	2	0.35 (0.04–2.77)	0.32	0.009, 0.85	R
		AA vs. AG/GG	1	0.75 (0.47–1.19)	0.22	-	F
		A vs. G	2	0.38 (0.06–2.58)	0.32	0.01, 0.84	R
	Japan	AA vs. GG	3	1.12 (0.87–1.45)	0.38	0.97, 0.00	F
		AG vs. GG	3	1.10 (0.95–1.28)	0.20	0.35, 0.5	R
		AA/AG vs. GG	3	1.10 (0.96–1.26)	0.15	0.40, 0.0	F
		AA vs. AG/GG	3	1.07 (0.84–1.37)	0.59	0.98, 0.0	F
		A vs. G	3	1.08 (0.97–1.20)	0.18	0.64, 0.0	F

^a^ Number of studies

^b^ P for OR

Co-dominant model; dominant model; recessive model; allelic contrast model

### Tests for publication bias and sensitivity analyses

**Fig 3:**
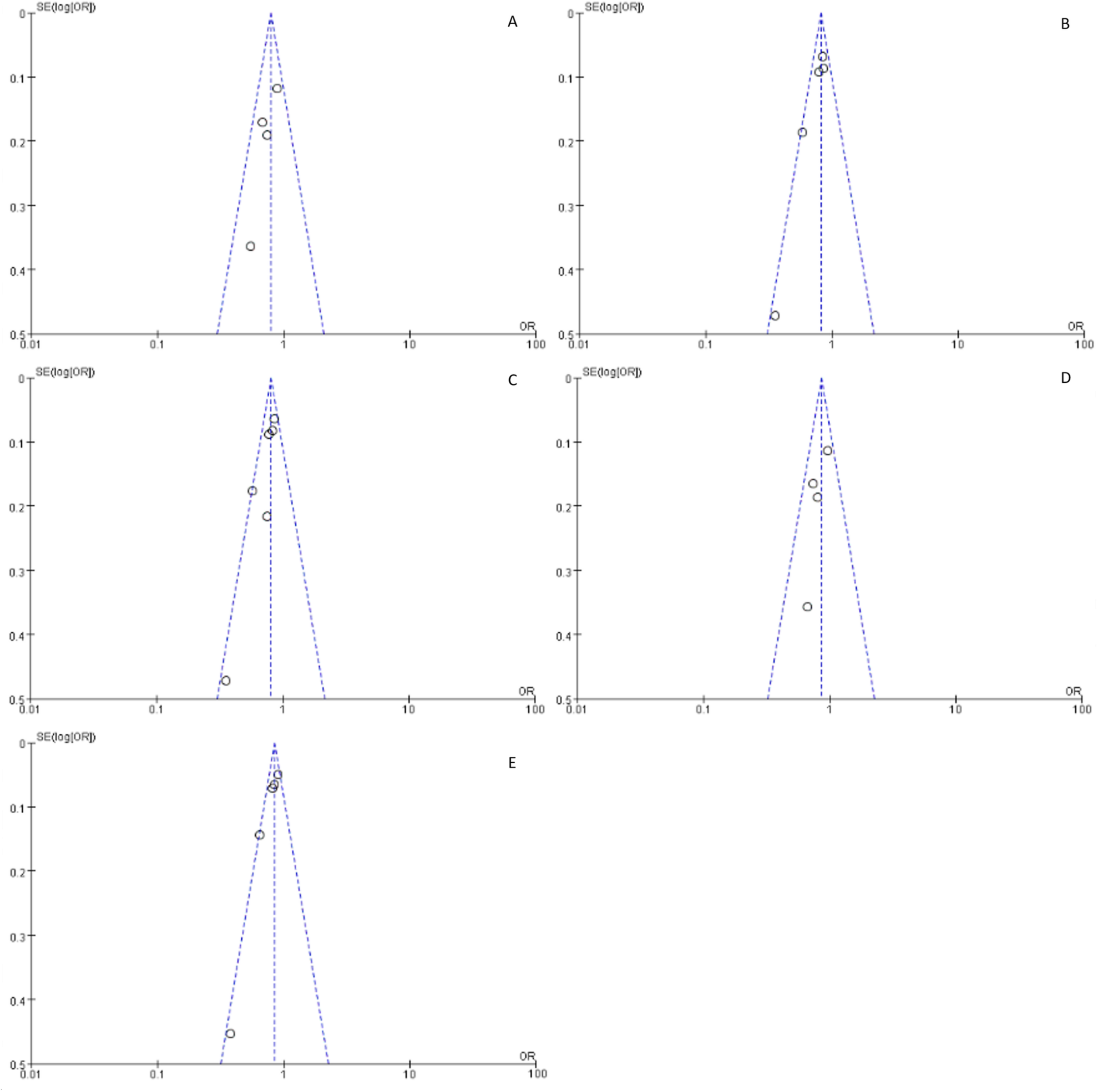
Funnel plot for association between *ALDH2* rs671 polymorphism and EH risk. (A) co-dominant model (AA vs. GG); (B) co-dominant model (AG vs. GG); (C) dominant model (AA/AG vs. GG); (D) recessive model (AA vs. AG/GG); (E) the allelic model (A vs. G).

**Fig 4:**
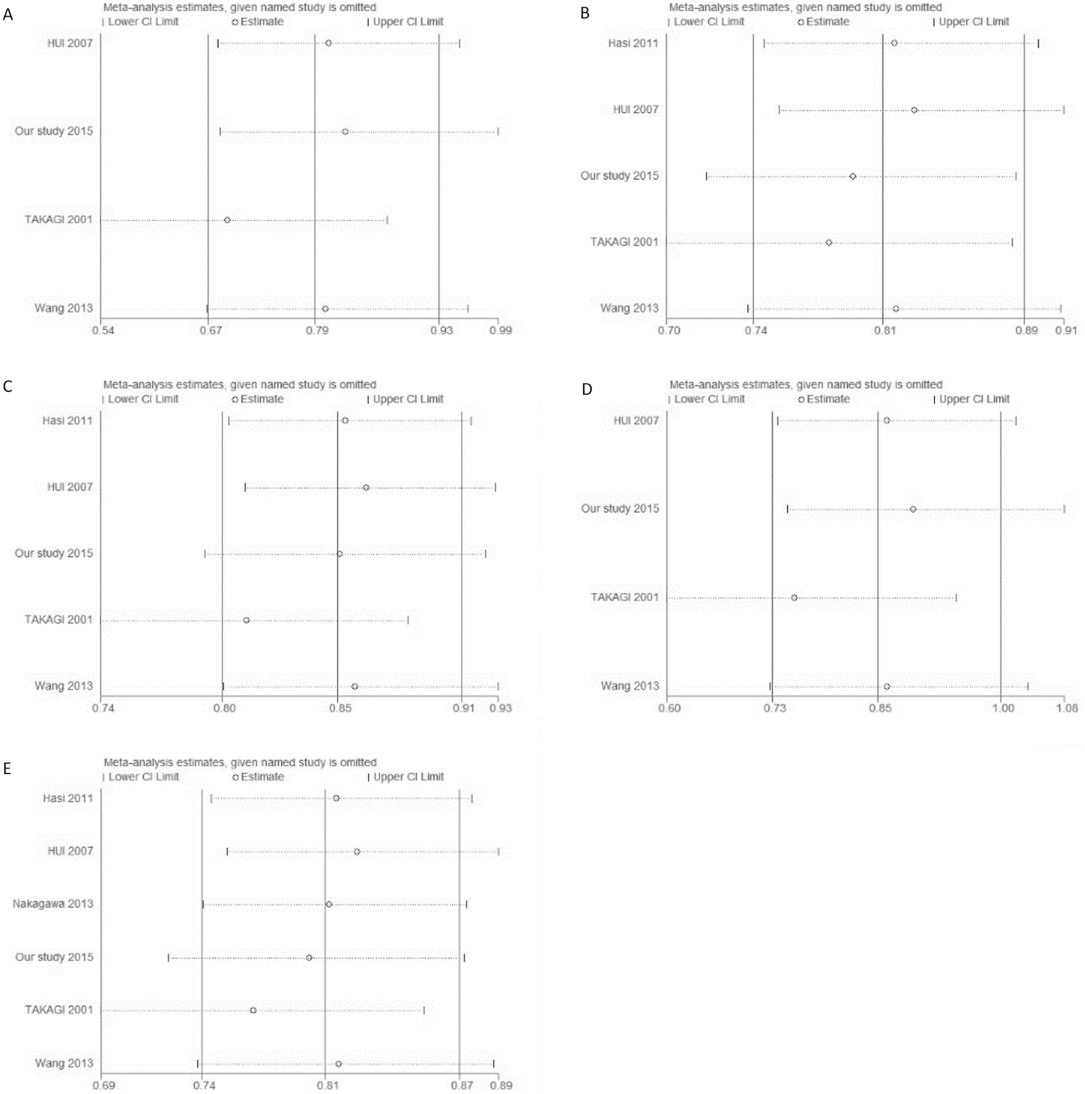
Sensitivity analysis of summary OR on association between *ALDH2* rs671 polymorphism and risk of EH. (A) co-dominant model (AA vs. GG); (B) co-dominant model (AG vs. GG); (C) dominant model (AA/AG vs. GG); (D) recessive model (AA vs. AG/GG); (E) allelic model (A vs. G).

Potential publication bias of this meta-analysis was detected by funnel plot (Fig 3), which revealed that there was no significant publication bias in any of the genetic models. Sensitivity analyses were conducted to detect the influence of each individual study on the pooled OR, with each study dataset being dropped one at a time. The outcomes did not vary greatly when any individual study was omitted, suggesting stability of the results (Fig 4).

## Discussion

Our results showed that the rs671 polymorphism in the *ALDH2* gene was significantly associated with EH risk in the case-control study and meta-analysis. The genotype frequencies of *ALDH2* rs671 polymorphism were 53.7% (GG), 40.3% (AG) and 6.0% (AA) in the cases, and 49.1% (GG), 43.0% (AG) and 7.9% (AA) in the controls in our case-control study. A significant association between the rs671 polymorphism and EH risk can be proved in all genetic models for Chinese cases and controls. This comprehensive analysis of different genetic models improves the accuracy of prediction and reduces the bias of single model prediction. Subgroup meta-analysis by country indicated a decreased risk of EH risk in the dominant model (AA/AG versus GG, OR = 0.75, 95% CI = 0.58–0.96) for Japanese cases and controls. Besides, the association between rs671 polymorphism and EH risk remained significant in all genetic models overall and in Japanese male subjects. We only found a significant association under the allelic contrast model among Chinese male subjects (A vs. G, OR = 0.82, 95% CI = 0.68–0.99), and no significant association between rs671 polymorphism and EH risk was found in women. Thus, we concluded that the GG genotype of rs671 appears to be associated with an increased risk of EH only in male subjects, especially in Japanese male subjects.

Mitochondrial *ALDH2* is responsible for the metabolism of toxic aldehydes [29].Individuals carrying inactive *ALDH2* may have a lower risk of alcohol-induced high blood pressure than people with the wild-type enzyme, who can consume more alcohol without experiencing acetaldehydemia [22]. Several studies suggested that *ALDH2* could reduce ROS-induced vascular contraction in angiotensin-II (AngII) hypertensive mice. And *ALDH2* protected both the microvasculature and microvasculature against reactive aldehydes generated under the condition of sustained oxidative stress [24, 30, 31]. Because of its ability to reduce the accumulation of acetaldehyde and the generation of reactive oxygen species (ROS), the rs671 GG genotype could be associated with a lower incidence of hypertension. Our case-control study showed that the *ALDH2* rs671 polymorphism was significantly associated with EH risk. Besides, the results showed that alcohol consumption was not a risk factor for EH, for there was no significant difference of drinking habit between cases and controls (P = 0.583). Thus, we concluded that the rs671 polymorphism of *ALDH2* gene was likely to be an independent risk factor.

A recent meta-analysis was published and also reported that the GG genotype of the rs671 polymorphism was a risk factor for EH [19]. However it was not clear whether or not the rs671 polymorphism is an independent risk factor of EH. Our study suggested that the rs671 polymorphism may be an independent risk factor according to the result of the case-control study. The association between the rs671 polymorphism and EH risk was also confirmed by the following comprehensive meta-analysis. However, populations in different geographical areas should be favored for the future study because both meta-analyses were conducted in Asian populations.

A study has shown that the rs671 polymorphism is associated with EH risk among Mongolian women but not men [23]. Our results are contrary to those studies. We found that the rs671 polymorphism was significantly associated with risk of EH among male subjects in the case-control study but not in women. Our findings can be explained in part by the physiological differences between men and women. Lagranha et al. have found that the female heart has increased phosphorylation and *ALDH2* activity, which detoxifies ROS-producing aldehyde adducts [32]. Meanwhile, some studies have shown that female hormones may protect women from developing high blood pressure [33]. In addition, ethnic background may also have played a role in the study of Hasi et al., which found a significant increase in the incidence of hypertension in women carrying *ALDH2* rs671 polymorphism [23].

Our study had some limitations. First, all case-control studies and meta-analyses were conducted in China and Japan, therefore, our findings might be applicable only to Asian populations. Second, due to the lack of uniform background data for studies included in meta-analysis, the data were not further stratified by other factors such as age, smoking, alcohol consumption and other lifestyle factors.

In conclusion, this study provides evidence that the rs671 GG genotype may influence the risk of EH independently of alcohol consumption, and mainly affects male subjects. Further investigations with larger sample sizes and detailed gene–environment data should be carried out to confirm these results, to provide an evidence base for public health management of EH.

## Supporting information

S1 Table Quality assessment of included studies. (DOCX)

S2 Table Meta-analysis on genetic association studies checklist. (DOCX)

S3 Table PRISMA checklist. (DOC)

S1 Text List of excluded citations and reasons. (DOCX)

## References

[1] He J, Gu D, Wu X, Reynolds K, Duan X, Yao C, et al Major Causes of Death among Men and Women in China. New England Journal of Medicine. 2005; 353(11):1124–34. 10.1056/NEJMsa050467 16162883

[2] Kakar P, Lip GY. Towards understanding the aetiology and pathophysiology of human hypertension: where are we now? Journal of Human Hypertension. 2006; 20(11): 833–36. Epub:2006/08/24. 10.1038/sj.jhh.1002082 16929340

[3] Gu DF, Jiang H, Wu XG. Prevalence, awareness, treatment and control of hypertension in Chinese adults. Zhonghua yu fang yi xue za zhi [Chinese journal of preventive medicine]. 2003; 37(2): 84–9. 12839656

[4] Kato N, Takeuchi F, Tabara Y, Kelly TN, Go MJ, Sim X, et al Meta-analysis of genome-wide association studies identifies common variants associated with blood pressure variation in east Asians. Nature Genetics. 2011; 43(6): 531–38. Epub 2011/05/15. PubMed Central PMCID:PMC3158568. 10.1038/ng.834 21572416 PMC3158568

[5] Fuchs FD, Chambless LE, Whelton PK, Nieto FJ, Heiss G. Alcohol consumption and the incidence of hypertension: The Atherosclerosis Risk in Communities Study. Hypertension. 2001; 37(5):1242–50. 11358935 10.1161/01.hyp.37.5.1242

[6] Krzesinski JM, Saint-Remy A. [Essential hypertension, a complex trait]. Revue Médicale De Liège. 2012; 67(67): 279–85. 22891479

[7] Ehret GB, Ferreira T, Chasman DI, Jackson AU, Schmidt EM, Johnson T, et al The genetics of blood pressure regulation and its target organs from association studies in 342,415 individuals. Nat Genet. 2016; 48(10): 1171–84. Epub 2016/09/12. 10.1038/ng.3667 27618452 PMC5042863

[8] Chen L, Smith GD, Harbord RM, Lewis SJ. Alcohol Intake and Blood Pressure: A Systematic Review Implementing a Mendelian Randomization Approach. Plos Medicine. 2008; 5(3):461–71. PubMed Central PMCID:PMC2265305. 10.1371/journal.pmed.0050052 PMC2265305 18318597

[9] Chang YC, Chiu YF, Lee IT, Ho LT, Hung YJ, Hsiung CA, et al Common ALDH2 genetic variants predict development of hypertension in the SAPPHIRe prospective cohort: Gene-environmental interaction with alcohol consumption. BMC Cardiovascular Disorders. 2012; 12(1):1–7. PubMed Central PMCID:PMC3476438. 22839215 10.1186/1471-2261-12-58 PMC3476438

[10] Takeshita T, Morimoto K, Mao XQ, Hashimoto T, Furuyama J. Phenotypic differences in low Kmaldehyde dehydrogenase in Japanese workers. Lancet. 1993; 341(8848):837–38. 8096045

[11] Perez-Miller S, Younus H, Vanam R, Chen CH, Mochly-Rosen D, Hurley TD. Alda-1 is an agonist and chemical chaperone for the common human aldehyde dehydrogenase 2 variant. Nature Structural & Molecular Biology. 2010; 17(2):159–64. Epub:2010/02/10. PubMed Central PMCID:PMC2857674. 10.1038/nsmb.1737 PMC2857674 20062057

[12] Harada S, Agarwal DP, Goedde HW, Tagaki S, Ishikawa B. Possible protective role against alcoholism for aldehyde dehydrogenase isozyme deficiency in Japan. Lancet. 1982; 2(8302): 827 6126701 10.1016/s0140-6736(82)92722-2

[13] Amamoto K, Okamura T, Tamaki S, Kita Y, Tsujita Y, Kadowaki T, et al Epidemiologic study of the association of low-Km mitochondrial acetaldehyde dehydrogenase genotypes with blood pressure level and the prevalence of hypertension in a general population. Clarendon Press. 2002; 25(6):857–64. 10.1291/hypres.25.857 12484509

[14] Takagi S, Baba S, Iwai N, Fukuda M, Katsuya T, Higaki J, et al The aldehyde dehydrogenase 2 gene is a risk factor for hypertension in Japanese but does not alter the sensitivity to pressor effects of alcohol: the Suita study. Hypertension Research Official Journal of the Japanese Society of Hypertension. 2001; 24(4): 365–70. 11510748 10.1291/hypres.24.365

[15] Hashimoto Y, Nakayama T, Futamura A, Omura M, Nakarai H, Nakahara K. Relationship between genetic polymorphisms of alcohol-metabolizing enzymes and changes in risk factors for coronary heart disease associated with alcohol consumption. Clinical Chemistry. 2002; 48(7): 4147–50. 12089173

[16] Levy D, Ehret GB, Rice K, Verwoert GC, Launer LJ, Dehghan A, et al Genome-wide association study of blood pressure and hypertension. Nature Genetics. 2009; 41(6): 677–87. Epub 2014/10/ 23. PubMed Central PMCID:PMC4303798. 10.1038/ng.384 19430479 PMC2998712

[17] Hao PP, Xue L, Wang XL, Chen YG, Wang JL, Ji WQ, et al Association between aldehyde dehydrogenase 2 genetic polymorphism and serum lipids or lipoproteins: A meta-analysis of seven East Asian populations. Atherosclerosis. 2010; 212(212): 213–16. Epub 2010/05/24. 20541757 10.1016/j.atherosclerosis.2010.05.024

[18] Zhang SY, Chan SW, Zhou X, Chen XL, Mok DKW, Lin ZX, et al Meta-analysis of association between ALDH2 s671 polymorphism and essential hypertension in Asian populations. Herz Supplement. 2015; 40:203–08. 10.1007/s00059-014-4166-2 25403981

[19] Jia K, Wang H, Dong P. Aldehyde dehydrogenase 2 (ALDH2) Glu504Lys polymorphism is associated with hypertension risk in Asians: a meta-analysis. International Journal of Clinical & Experimental Medicine. 2015; 8(7):10767–72. PubMed Central PMCID:PMC4565253. 26379870 PMC4565253

[20] Moher D, Liberati A, Tetzlaff J, Altman DG; PRISMA Group. Reprint preferred reporting items for systematic reviews and meta-analyses: the PRISMA statement. Physical Therapy. 2009; 89(9): 873–80. 19723669

[21] Little J, Higgins JP, Ioannidis JP, Moher D, Gagnon F, von Elm E, et al STrengthening the REporting of Genetic Association Studies (STREGA): an extension of the STROBE statement. PLoS medicine. 2009; 6(2):e22 PubMed Central PMCID: PMC2730482. 10.1371/journal.pmed.1000022 19192942 PMC2634792

[22] Peng GS, Chen YC, Tsao TP, Wang MF, Yin SJ. Pharmacokinetic and pharmacodynamic basis for partial protection against alcoholism in Asians, heterozygous for the variant ALDH2*2 gene allele. Pharmacogenetics & Genomics. 2007; 17(10): 845–55. 17885622 10.1097/FPC.0b013e3282609e67

[23] Hasi T, Hao L, Yang L, Su XL. Acetaldehyde dehydrogenase 2 SNP rs671 and susceptibility to essential hypertension in Mongolians: a case control study. Genetics & Molecular Research Gmr. 2011; 10(1): 537–43. 21476199 10.4238/vol10-1gmr1056

[24] Nakagawa T, Kajiwara A, Saruwatari J, Hamamoto A, Kaku W, Oniki K, et al The combination of mitochondrial low enzyme-activity aldehyde dehydrogenase 2 allele and superoxide dismutase 2 genotypes increases the risk of hypertension in relation to alcohol consumption. Pharmacogenetics & Genomics. 2013; 23(1):18323–36. 10.1097/FPC.0b013e32835b1707 23111423

[25] Wang Y, Zhang Y, Zhang J, Tang X, Qian Y, Gao P, et al Association of a functional single-nucleotide polymorphism in the ALDH2 gene with essential hypertension depends on drinking behavior in a Chinese Han population. Journal of Human Hypertension. 2013; 27(3): 181–6. Epub 2012/05/03. 10.1038/jhh.2012.15 22551939

[26] Yokoyama A, Mizukami T, Matsui T, Yokoyama T, Kimura M, Matsushita S, et al Genetic polymorphisms of alcohol dehydrogenase-1B and aldehyde dehydrogenase-2 and liver cirrhosis, chronic calcific pancreatitis, diabetes mellitus, and hypertension among Japanese alcoholic men. Alcoholism: Clinical and Experimental Research. 2013; 37(8): 1391–01. Epub 2013.03/29. 10.1111/acer.12108 23550892

[27] Ma X, Zheng S, Shu Y, Wang Y, Chen X. Association of the Glu504Lys polymorphism in the aldehyde dehydrogenase 2 gene with endothelium-dependent dilation disorder in Chinese Han patients with essential hypertension. Internal Medicine Journal. 2016; 46(5): 608–15. 10.1111/imj.12983 26691593

[28] Ota M, Hisada A, Lu X, Nakashita C, Masuda S, Katoh T. Associations between aldehyde dehydrogenase 2 (ALDH2) genetic polymorphisms, drinking status, and hypertension risk in Japanese adult male workers: a case-control study. Environmental Health and Preventive Medicine. 2016; 21(1): 1–8. Epub 2015/08/ 30. PubMed Central PMCID:PMC4693762[Available on 2017-01-01]. 10.1007/s12199-015-0490-2 26318866 PMC4693762

[29] Jaubert MP, Jin Z, Russo C, Schwartz JE, Homma S, Elkind MS, et al Alcohol consumption and ambulatory blood pressure: a community-based study in an elderly cohort. American Journal of Hypertension. 2014; 27(5): 688–94. Epub 2013/12/21. PubMed Central PMCID:PMC3978947. 10.1093/ajh/hpt235 24363276 PMC3978947

[30] Saito K, Yokoyama T, Yoshiike N, Date C, Yamamoto A, Muramatsu M, et al Do the ethanol metabolizing enzymes modify the relationship between alcohol consumption and blood pressure? Journal of Hypertension. 2003; 21(6): 1097–105. 10.1097/01.hjh.0000059045.65882.92 12777946

[31] Hiura Y, Tabara Y, Kokubo Y, Okamura T, Miki T, Tomoike H, et al A genome-wide association study of hypertension-related phenotypes in a Japanese population. Circulation Journal Official Journal of the Japanese Circulation Society. 2010; 74(11): 2353–59. 20877124 10.1253/circj.cj-10-0353

[32] Lagranha CJ, Deschamps A, Aponte A, Steenbergen C, Murphy E. Sex differences in the phosphorylation of mitochondrial proteins result in reduced production of reactive oxygen species and cardioprotection in females. Circulation Research. 2010; 106(11): 1681–91. Epub 2010/05/22. PubMed Central PMCID:PMC3127199. 10.1161/CIRCRESAHA.109.213645 20413785 PMC3127199

[33] Weiner CP, Lizasoain I, Baylis SA, Knowles RG, Charles IG, Moncada S. Induction of calcium-dependent nitric oxide synthases by sex hormones. Proceedings of the National Academy of Sciences of the United States of America.1994; 91(11): 5212–16. PubMed Central PMCID:PMC43962. 7515189 10.1073/pnas.91.11.5212 PMC43962

