# Supplementary material for: Positive association between ALDH2 rs671 polymorphism and essential hypertension: A case-control study and meta-analysis
Source: PLoS One. 2017 May 4;12(5):e0177023. doi: 10.1371/journal.pone.0177023 (PMC5417637; doi:10.1371/journal.pone.0177023)
Supplement: S1 Table — (DOCX) [file pone.0177023.s001.docx]

S2 Table Quality assessment of included studies

| Last  name of  first  author | Year | Clear  description of  background,  objectives and  study design | Clear  eligibility  criteria | Clear  Definitionof  variables | Credible  genotyping  methods | Hardy-  Weinberg  equilibrium  assessment | Clear  Descriptionof statistical  methods | Summary of  characteristics  of participants | Publicly  available  genotype  data | Comprehensive  discussion |
| --- | --- | --- | --- | --- | --- | --- | --- | --- | --- | --- |
| Ota | 2016 | + | + | + | + | - | + | + | + | + |
| Ma | 2015 | + | + | + | + | + | + | + | + | + |
| Nakagawa | 2013 | + | + | + | + | - | + | ± | + | + |
| Yokoyama | 2013 | + | + | + | + | - | + | + | + | + |
| Wang | 2013 | + | + | + | + | + | + | + | + | + |
| Hasi | 2011 | + | + | + | + | - | + | ± | + | + |
| Hui | 2007 | + | + | + | + | + | + | + | + | + |
| Amamoto | 2002 | + | + | + | + | - | + | + | + | + |
| Takagi | 2001 | + | + | + | + | - | + | + | + | + |
| Our study | 2015 | + | + | + | + | + | + | + | + | + |

*: “+”: detailed description; “±”: incomplete description; “-”: no description.
