# Supplementary material for: Positive association between ALDH2 rs671 polymorphism and essential hypertension: A case-control study and meta-analysis
Source: PLoS One. 2017 May 4;12(5):e0177023. doi: 10.1371/journal.pone.0177023 (PMC5417637; doi:10.1371/journal.pone.0177023)
Supplement: S1 Text — (DOCX) [file pone.0177023.s004.docx]

S1 Text. List of excluded citations and reasons

**1. 35 Citations excluded through screening of titles and abstracts.**

1. Okayama A, Ueshima H, Yamakawa M, Kita Y. Low-Km aldehyde dehydrogenase deficiency does not influence the elevation of blood pressure by alcohol. J Hum Hypertens. 1994;8(3):205–08.PMID: 8006921.

2. Itoh T, Matsumoto M, Nakamura M, Okada A, Shirahashi N,Hougaku H,et al. Effects of daily alcohol intake on the blood pressure differ depending on an individual's sensitivity to alcohol: oriental flushingas a sign to stop drinking for health reasons. Journal of Hypertension. 1997; 15(11): 1211–17. PMID:9383169.

3. Shibata A, Fukuda K, Nishiyori A, Ogimoto I, Sakata R, Tanikawa K. A case-control study on male hepatocellular carcinoma based on hospital and community controls. Journal of epidemiology / Japan Epidemiological Association.1998; 8(1):1–5. PMID: 9575688.

4. Amamoto K, Okamura T, Tamaki S, Kita Y, Tsujita Y,Kadowaki T,et al. Epidemiologic study of the association of low-Km mitochondrial acetaldehyde dehydrogenase genotypes with blood pressure level and the prevalence of hypertension in a general population. Clarendon Press. 2002; 25(6):857–64. PMID:12484509.

5. Yamada Y, Sun F, Tsuritani I, Honda R. Genetic differences in ethanol metabolizing enzymes and blood pressure in Japanese alcohol consumers. J Hum Hypertens. 2002;16(7):479–86. doi: 10.1038/sj.jhh.1001415. PMID: 12080432.

6. Murata C, Watanabe T, Furuya H, Sugioka Y, Mikurube H, Yokoyama A, et al. Aldehyde dehydrogenase 2 and beta3-adrenergic receptor gene polymorphisms: their association with elevated liver enzymes and metabolic syndrome. Metabolism: clinical and experimental. 2003; 52(9):1096–101.PMID: 14506613.

7. Ohsawa I, Kamino K, Nagasaka K, Ando F, Niino N, Shimokata H, et al. Genetic deficiency of a mitochondrial aldehyde dehydrogenase increases serum lipid peroxides in community-dwelling females. Journal of human genetic. 2003, 48(8):404–09. Epub 2003/08/7. doi: 10.1007/s10038-003-0046-y. PMID: 12905081.

8. Saito K, Yokoyama T, Yoshiike N, Date C, Yamamoto A, Muramatsu M, et al. Do the ethanol metabolizing enzymes modify the relationship between alcohol consumption and blood pressure? Journal of hypertensio. 2003; 21(6):1097–105. doi:10.1097/01.hjh.0000059045.65882.92.PMID: 12777946.

9. Yamada Y. Alcohol consumption as a risk factor in the development of hypertension. Nihon rinsho Japanese journal of clinical medicine. 2004; 62(3):70–74.PMID: 15171344.

10. Moiseev VS, Ogurtsov PP, Kobalova Zh D, Ovchinnikov NS, Garmash IV, Mazurchik NV, et al. Alcohol-induced arterial hypertension and genetic polymorphism of alcohol-metabolizing enzymes. Terapevticheskii arkhiv. 2005; 77(6):54–60.PMID: 16078602.

11. Nishiyori A, Shibata A, Ogimoto I, Uchimura N, Egami H, Nakamura J, et al. Alcohol drinking frequency is more directly associated with alcohol use disorder than alcohol metabolizing enzymes among male Japanese. Psychiatry and clinical neurosciences. 2005; 59(1):38–44. doi: 10.1111/j.1440-1819.2005.01329.x. PMID: 15679538.

12. Yamamoto T, Moriwaki Y, Takahashi S. Effect of ethanol on metabolism of purine bases (hypoxanthine, xanthine, and uric acid). Clinica chimica acta; international journal of clinical chemistry. 2005; 356(1–2):35–57. Epub 2005/03/29. doi: 10.1016/j.cccn.2005.01.024. PMID: 15936302.

13. Hui P, Nakayama T, Morita A, Sato N, Hishiki M, Saito K,et al.Common single nucleotide polymorphisms in Japanese patients with essential hypertension: aldehyde dehydrogenase 2 gene as a risk factor independent of alcohol consumption. Hypertension Research. 2007; 30(7): 585–92. doi:[10.1291/hypres.30.585](https://dx.doi.org/10.1291/hypres.30.585) PMID:17785925.

14. Jo SA, Kim EK, Park MH, Han C, Park HY, Jang Y, et al. A Glu487Lys polymorphism in the gene for mitochondrial aldehyde dehydrogenase 2 is associated with myocardial infarction in elderly Korean men. Clinica Chimica Act. 2007; 382(1–2):43–47. Epub 2007/03/24.PMID: 17459359.

15. Nagasawa H, Wada M, Arawaka S, Kawanami T, Kurita K, Daimon M, et al. A polymorphism of the aldehyde dehydrogenase 2 gene is a risk factor for multiple lacunar infarcts in Japanese men: the Takahata Study. European Journal of Neurology. 2007; 14(4):428–34. doi: 10.1111/j.1468-1331.2007.01700.x. PMID: 17388993.

16. Tsuchihashi-Makaya M, Serizawa M, Yanai K, Katsuya T, Takeuchi F, Fujioka A, et al. Gene-environmental interaction regarding alcohol-metabolizing enzymes in the Japanese general population. Hypertension research : official journal of the Japanese Society of Hypertension. 2009; 32(3):207–13. Epub 2009/02/20. doi: 10.1038/hr.2009.3. PMID: 19262484.

17. Hiura Y, Tabara Y, Kokubo Y, Okamura T, Miki T, [Tomoike H](https://www.ncbi.nlm.nih.gov/pubmed/?term=Tomoike%20H%5bAuthor%5d&cauthor=true&cauthor_uid=20877124),et al. A genome-wide association study of hypertension-related phenotypes in a Japanese population. Circulation Journal Official Journal of the Japanese Circulation Society. 2010; 74(11): 2353–59. PMID:20877124.

18. Choi H, Tostes RC, Webb RC. Mitochondrial aldehyde dehydrogenase prevents ROS-induced vascular contraction in angiotensin-II hypertensive mice. Journal of the American Society of Hypertension : JASH. 2011; 5(3):154–60. Epub 2011/04/01. doi: 10.1016/j.jash.2011.02.005. PMID: 21459068; PubMed Central PMCID: PMC3085594.

19. Yao CT, Cheng CA, Wang HK, Chiu SW, Chen YC, Wang MF, et al. The role of ALDH2 and ADH1B polymorphism in alcohol consumption and stroke in Han Chinese. Human genomics. 2011; 5(6):569–76.PMID: 22155604; PubMed Central PMCID: PMC3525250.

20. Jung JG, Kim JS, Yoon SJ, Oh MK. Relationships Among Alcohol Consumption, Facial Flushing Response, and Metabolic Syndrome in Healthy Men. Annals of Epidemiology. 2012; 22(7):480–86. Epub 2012 May 9. doi: 10.1016/j.annepidem.2012.04.014.PMID: 22575812.

21. Lai CL, Liu MT, Yin SJ, Lee JT, Lu CC, Peng GS. Heavy binge drinking may increase risk of stroke in nonalcoholic hypertensives carrying variant ALDH2*2 gene allele. Acta neurologica Taiwanica. 2012;21(1):39–43.PMID: 22879089.

22. Lai X, Wang J, Nabar NR, Pan S, Tang C, Huang Y, et al. Proteomic response to acupuncture treatment in spontaneously hypertensive rats. PLoS One. 2012; 7(9): e44216. Epub 2012/09/12. doi: 10.1371/journal.pone.0044216.PMID: 22984478; PubMed Central PMCID: PMC3440387.

23. Morita K, Saruwatari J, Miyagawa H, Uchiyashiki Y, Oniki K, Sakata M,et al. Association between aldehyde dehydrogenase 2 polymorphisms and the incidence of diabetic retinopathy among Japanese subjects with type 2 diabetes mellitus. Cardiovascular Diabetology. 2013;12. doi: 10.1186/1475-2840-12-132.PMID: 24028448 PubMed Central PMCID: PMC3847457.

24. Seok H, Yoo KH, Kim YO, Chung JH. Association of a Missense ALDH2 Single Nucleotide Polymorphism (Glu504Lys) With Benign Prostate Hyperplasia in a Korean Population. International Neurourology Journal. 2013; 17(4):168–73. Epub 2013/12/31. doi: 10.5213/inj.2013.17.4.168. PMID: 24466463; PubMed Central PMCID: PMC3895508.

25. Wang H, Pan Q, Gao Q, Kang P, Li M, He P, et al. Correlation of aldehyde dehydrogenase-2 gene polymorphism with hypertension in patients with coronary heart disease complicated by diabetes mellitus. Nan fang yi ke da xue xue bao = Journal of Southern Medical University. 2013;33(4):542–45, 562.PMID: 23644116.

26. Hu N, Zhang Y, Nair S, Culver BW, Ren J. Contribution of ALDH2 polymorphism to alcoholism-associated hypertension. Recent patents on endocrine, metabolic & immune drug discovery. 2014; 8(3):180–85.PMID: 25354396.

27. Matsumoto A, Vasiliou V, Kawamoto T, Tanaka K, Ichiba M. Ethanol Reduces Lifespan, Body Weight, and Serum Alanine Aminotransferase Level of Aldehyde Dehydrogenase 2 Knockout Mouse. Alcoholism-Clinical and Experimental Research. 2014;38(7):1883–93. Epub 2014/06/13. doi: 10.1111/acer.12462.PMID: 24930774.

28. Morita K, Oniki K, Miyazaki H, Saruwatari J, Ogata Y, Mizobe M, et al. Aldehyde dehydrogenase 2 as a potential protective factor for renal insufficiency in Japanese subjects with heart failure: a pilot study. Journal of Human Hypertension. 2014; 28(4):279–81. Epub 2013/09/26. doi: 10.1038/jhh.2013.90. PMID: 24067347.

29. Sonoda K, Ohtake K, Kubo Y, Uchida H, Uchida M, Natsume H, et al. Aldehyde dehydrogenase 2 partly mediates hypotensive effect of nitrite on L-NAME-induced hypertension in normoxic rat. Clinical and Experimental Hypertension. 2014; 36(6):410–18. Epub 2013/10/28 doi: 10.3109/10641963.2013.846355. PMID: 24164360.

30. Xu F, Sun Y, Shang R, Li M, Cui L, Cui Z, Chen Y. The Glu504Lys Polymorphism of Aldehyde Dehydrogenase 2 Contributes to Development of Coronary Artery Disease. Tohoku Journal of Experimental Medicine. 2014; 234(2):143–50.PMID: 25263942.

31. Zhang Y, Mi SL, Hu N, Doser TA, Sun A, Ge J, et al. Mitochondrial aldehyde dehydrogenase 2 accentuates aging-induced cardiac remodeling and contractile dysfunction: role of AMPK, Sirt1, and mitochondrial function. Free Radical Biology and Medicine. 2014; 71:208–20. Epub 2014/03/24. doi: 10.1016/j.freeradbiomed.2014.03.018. PMID: 24675227; PubMed Central PMCID: PMC4068748.

32. Campos JC, Fernandes T, Grassmann Bechara LR, da Paixao NA, Brum PC, de Oliveira EM, et al. Increased Clearance of Reactive Aldehydes and Damaged Proteins in Hypertension-Induced Compensated Cardiac Hypertrophy: Impact of Exercise Training. Oxidative Medicine and Cellular Longevity. 2015. Epub 2015/04/14.doi: 10.1155/2015/464195. PMID: 25954323;PubMed Central PMCID: PMC4411445.

33. Liu JF, Xia P, Hu WQ, Wang D, Xu XY. Aldehyde dehydrogenase 1 expression correlates with clinicopathologic features of patients with breast cancer: a meta-analysis. International journal of clinical and experimental medicine. 2015; 8(6):8425–32.PMID: 26309495;PubMed Central PMCID: PMC4537955.

34. Lu X, Wang L, Lin X, Huang J, Charles Gu C, He M, et al. Genome-wide association study in Chinese identifies novel loci for blood pressure and hypertension. Human molecular genetics. 2015; 24(3):865–74. Epub 2014/09/23.doi: 10.1093/hmg/ddu478.PMID: 25249183; PubMed Central PMCID: PMC4303798.

35. Shen C, Wang C, Fan F, Yang Z, Cao Q, Liu X, et al. Acetaldehyde dehydrogenase 2 (ALDH2) deficiency exacerbates pressure overload-induced cardiac dysfunction by inhibiting Beclin-1 dependent autophagy pathway. Biochimica Et Biophysica Acta-Molecular Basis of Disease. 2015;1852(2):310–18.Epub 2014 Jul 30. doi: 10.1016/j.bbadis.2014.07.014. PMID: 25086229.

**2. 8 studies excluded for not case-control studies.**

1. Ishikawa N, Murata C, Mikurube H, Ito S, Higashiyama R, Komaki Y, et al. Change of components of the metabolic syndrome in a workers' health checkup after five years--relation with elevated liver enzymes, gene polymorphisms for ALDH 2, beta3-AR and lifestyle. [Nihon koshu eisei zasshi] Japanese journal of public health. 2005; 52(11):979–86.PMID:16408483.

2. Chen L, Smith GD, Harbord RM, Lewis SJ. Alcohol Intake and Blood Pressure: A Systematic Review Implementing a Mendelian Randomization Approach. Plos Medicine. 2008; 5(3):461–71. doi:10.1371/journal. pmed.0050052 PMID:18318597; PubMed Central PMCID:[PMC2265305](https://www.ncbi.nlm.nih.gov/pmc/articles/PMC2265305/).

3. Yin R, Wu J, Pan S, Lin W, Yang D, Chen Y. Sex differences in environmental and genetic factors for hypertension. American Journal of Medicine. 2008; 121(9):811–19. doi: 10.1016/j.amjmed.2008.04.026.PMID: 18724972.

4. Hao PP, Xue L, Wang XL, Chen YG, Wang JL, Ji WQ, et al. Association between aldehyde dehydrogenase 2 genetic polymorphism and serum lipids or lipoproteins: A meta-analysis of seven East Asian populations. Atherosclerosis. 2010; 212(212): 213–16. Epub 2010/05/24. doi:10.1016/j.atherosclerosis.2010.05.024 PMID: 20541757.

5. Chang YC, Chiu YF, Lee IT, Ho LT, Hung YJ,Hsiung CA, et al. Common ALDH2 genetic variants predict development of hypertension in the SAPPHIRe prospective cohort: Gene-environmental interaction with alcohol consumption. BMC Cardiovascular Disorders. 2012; 12(1):1–7. doi: [10.1186/1471-2261-12-58](https://dx.doi.org/10.1186/1471-2261-12-58) PMID:22839215; PubMed Central PMCID:[PMC3476438](https://www.ncbi.nlm.nih.gov/pmc/articles/PMC3476438/).

6. Isomura M, Wang T, Yamasaki M, Hasan MZ, Shiwaku K, Nabika T. Aldehyde Dehydrogenase Polymorphisms and Blood Pressure Elevation in the Japanese: A Cross-Sectional and a Longitudinal Study over 20 Years in the Shimane CoHRE Study. Disease Markers. 2015;2015:825435. Epub 2015/06/22. doi: 10.1155/2015/825435. PMID: 26185357; PubMed Central PMCID: PMC4491569.

7. Jia K, Wang H, Dong P. Aldehyde dehydrogenase 2 (ALDH2) Glu504Lys polymorphism is associated with hypertension risk in Asians: a meta-analysis. International Journal of Clinical & Experimental Medicine. 2015; 8(7):10767–72. PMID:26379870; PubMed Central PMCID:[PMC4565253](https://www.ncbi.nlm.nih.gov/pmc/articles/PMC4565253/).

8. Chang YC, Chiu YF, Lee IT, Ho LT, Hung YJ,Hsiung CA, et al. Common ALDH2 genetic variants predict development of hypertension in the SAPPHIRe prospective cohort: Gene-environmental interaction with alcohol consumption. BMC Cardiovascular Disorders. 2012; 12(1):1–7. doi: [10.1186/1471-2261-12-58](https://dx.doi.org/10.1186/1471-2261-12-58) PMID:22839215; PubMed Central PMCID:[PMC3476438](https://www.ncbi.nlm.nih.gov/pmc/articles/PMC3476438/).

**3. 1 studies excluded for not providing sufficient data, though we have tried to contact authors.**

1. Zhang WS, Xu L, Schooling CM, Jiang CQ, Cheng KK, Liu B, et al. Effect of alcohol and aldehyde dehydrogenase gene polymorphisms on alcohol-associated hypertension: the Guangzhou Biobank Cohort Study. Hypertension research : official journal of the Japanese Society of Hypertension. 2013; 36(8):741–46. Epub 2013/04/25. doi: 10.1038/hr.2013.23 PMID: 23615284; PubMed Central PMCID: PMC3734527.
